# The Role of Artificial Intelligence in the Diagnosis and Management of Non-convulsive Seizures: A Narrative Review

**DOI:** 10.7759/cureus.79409

**Published:** 2025-02-21

**Authors:** Aishwarya Gadwal, Maria Gabriela Cerdas, Areej Khan, Zainab Khan, Di Carluccio Martina, Sai Priya Nimmagadda, Syed Mustafa Ali Rizvi, Sara Mahmood Aljallawi, Shelly Jaryal, Nuren Tasgaonkar, Adnan Ahmed, Sowmika Reddy Busireddy

**Affiliations:** 1 Radiology, Kasturba Medical College, Mangalore, IND; 2 General Medicine, Universidad de Ciencias Médicas (UCIMED), San José, CRI; 3 General Medicine, Indian Institute of Medical Science and Research, Warudi, IND; 4 General Medicine, Gajra Raja Medical College (GRMC), Gwalior, IND; 5 Neurology, Policlinico Campus Bio-Medico, Rome, ITA; 6 Internal Medicine, Gandhi Medical College, Secunderabad, IND; 7 Critical Care Medicine, B. L. Kapur-Max Super Speciality Hospital, New Delhi, IND; 8 General Medicine, Mansoura University, Mansoura, EGY; 9 Internal Medicine, Medical University of the Americas (MUA), Charlestown, KNA; 10 General Medicine, Shri Dharmasthala Manjunatheshwara (SDM) College of Medical Sciences and Hospital, Dharwad, IND; 11 Internal Medicine, York University, Toronto, CAN; 12 Internal Medicine, Malla Reddy Medical College for Women, Hyderabad, IND

**Keywords:** artificial intelligence, eeg analysis, machine learning, non-convulsive seizures, seizure management

## Abstract

Non-convulsive seizures (NCS) are often underdiagnosed due to their subtle presentation including changes in behavior and mental status. Although electroencephalography (EEG) remains the gold standard for detection, challenges, such as subjective interpretation, individual observer variability, and limited availability, often prolong diagnosis. This can lead to severe complications, including cognitive decline and higher mortality rates.

Recent developments in artificial intelligence (AI) are revolutionizing epilepsy care by providing enhanced accuracy and efficiency for diagnosing and managing NCS. Machine learning models, including convolutional neural networks (CNN), recurrent neural networks (RNN), and support vector machines (SVM) have demonstrated high precision in analyzing EEG data and predicting seizures. Innovations such as Ceribell Clarity algorithm (Ceribell, Sunnyvale, CA) allow fast, real-time seizure detection, reducing diagnostic delays in emergency and critical care. Wearable AI-driven technologies like wearable monitoring devices, predictive analytics, and explainable AI enhance personalized care and support better clinician decision-making.

This review underlines AI’s potential in neurology and neurosurgery, highlighting its role in enhancing diagnostic precision, accelerating interventions, and supporting surgical and treatment planning. By incorporating AI into clinical practice, healthcare systems can overcome diagnostic challenges and deliver patient-centered care. AI is becoming a key element in the future of medicine, driving advances in precision neurology and improving patient outcomes worldwide.

## Introduction and background

Seizures are defined as abnormal electrical activity in the brain and are a common neurological disorder that can manifest in various forms, including convulsive seizures, which are accompanied by visible motor activity, and non-convulsive seizures (NCS), which lack overt motor symptoms and are, therefore, more challenging to detect. The symptoms are often subtle, such as memory lapses, confusion, changes in speech, and altered mental status (AMS) [1,2]. Because these symptoms are subtle and overlap with other conditions such as delirium, psychiatric disorders, migraine with aura, metabolic encephalopathy, and sleep disorders [3], NCS is thus often misdiagnosed. Additionally, brain tumors can also present with seizures, particularly in cases where they disrupt normal neuronal activity, further complicating the diagnosis and treatment of NCS [2]. This misdiagnosis can delay proper treatment, leading to potentially serious outcomes, which in turn may delay proper treatment and potentially can lead to serious outcomes like brain injury, long-term cognitive impairments, and increased morbidity [4]. The challenge of detecting NCS is further compounded by the fact that some categories, such as "simple partial," "complex partial," and “absence seizures,” are only for a few seconds with minimal symptoms [5]. If left untreated, it can result in significant neurological consequences, including cognitive decline and long-term functional impairments [6].

To help detect NCS, at least 24 hours of continuous EEG (cEEG) is recommended [7]. Unfortunately, due to its high costs and the fact that it needs to be operated by specialized technicians, cEEG is not readily available at all places [6]. This often results in physicians relying on their clinical judgment, which may lead to mistreatment of patients [6]. Even with the right tools, diagnosis is still difficult as a physician must match the behavioral signs and the electrographic data from the EEG, but these patterns may not correspond with any visible changes in behavior [6]. Moreover, there may be underlying brain damage that can result in abnormal brain activity that would further complicate the interpretation of EEG readings [6]. Therefore, as the need for improved diagnostic accuracy grows, leveraging artificial intelligence (AI) in the diagnosis and management of NCS can potentially be a viable solution to overcome these challenges.

AI is described as a scientific and engineering discipline focused on understanding and replicating what is considered intelligent behavior through computational methods. It also involves developing systems capable of demonstrating such behavior [8]. In modern medicine, one of the significant challenges is managing, analyzing, and utilizing vast amounts of information required to address intricate clinical issues. AI seeks to emulate human cognitive abilities, driving a transformative shift in healthcare, supported by the growing availability of healthcare data and advancements in analytical techniques [8].

AI and machine learning (ML) are data-driven methodologies designed to transform raw data into meaningful and actionable insights, aiding clinical decision-making processes [8]. These technologies have shown highly promising early results, generating both enthusiasm and significant attention. This article explores the application of AI and ML in the context of NCS, aiming to provide practicing neurologists with an understanding of the advantages and limitations of incorporating these tools into clinical practice. Additionally, it addresses the practical, ethical, and equity-related considerations associated with the use of AI in medicine.

## Review

Overview of non-convulsive seizures 

NCS are abnormal electrical activity within the brain that does not cause motor activity like convulsive seizures [9]. Non-convulsive status epilepticus (NCSE) is a more serious form of NCS in which there is continuous seizure activity in the form of cognitive or behavioral changes for a minimum of 30 minutes [9]. Almost half of the NCS are in the form of NCSE [10]. Seizures affect one in 26 individuals worldwide, and more than 80% of people with epilepsy (PWE) live in low-middle-income countries [11]. A retrospective study done in an intensive care unit (ICU) setting has shown that almost 50% of patients with diagnosed status epilepticus were non-convulsive type [9]. 

Clinically, it most commonly presents as AMS in the form of confusion, lethargy, delirium, agitation, stupor, coma, even depression, or inappropriate behavior. AMS is a frequently witnessed issue in the hospital, around 5% of which is because of NCS [10,12]. Previous studies done on NCS have concluded that female sex, history of epilepsy or tonic-clonic seizures, and discontinued benzodiazepines for treatment contribute to the risk factors [10]. It has also been established that increasing age is associated with an increased risk of developing NCS and NCSE [10]. Additionally, the usual presentation has multiple differentials within this age group, making it further difficult to diagnose [13]. In a case series study of 22 elderly patients with AMS, including protracted confusion, reduced concentration and attention, speech disturbances, and subtle ictal manifestations had a delay of about five days to diagnose NCS/NCSE [14].

Electroencephalography (EEG) is the gold standard for diagnosing NCS. Patients with NCS commonly seek care in the emergency department, many of which do not have the EEG facility required to make the diagnosis. This delay can lead to fatal complications such as neuronal injury and cardiac arrhythmias [10]. Further challenges that arise with EEG diagnosis for seizures include patient behavior, approximation of electrodes during an unusual event, and appearance of artifacts due to poor placement of electrodes. Most importantly, EEG interpretation is very much subjective to the individual interpreting the findings. Even in countries with advanced healthcare systems, doctors or technicians without fellowship training in EEG interpret the findings. Without an objective numeric criterion for frequency, amplitude, morphology, and evolution of electrographic activity, it gets challenging on its own [9,11,15].

NCS and NCSE, when diagnosed in patients, are linked to longer hospital/ICU stays and increased mortality and morbidity [16]. Outcomes in non-ICU are usually good, and patients respond well to treatment when promptly diagnosed. NCSE can lead to structural abnormalities within the brain, causing persistent cognitive and memory deficits [9]. A study done on pediatric and young adult patients with NCSE showed that 31% of the patients had significant neurological morbidity on discharge [17]. 

AI in neurology

AI, along with any other system, does have its benefits and limitations. Some of the key benefits include improved diagnosis, enhanced disease prediction, clinical decision support, efficient data management, early intervention in neurodegenerative diseases, telemedicine, and remote monitoring [18]. Even with these advances in the use of AI in medicine, the quality of life has significantly improved in patients [18]. Being able to answer questions with good enough precision is one thing. However, along with benefits, there are limitations to these AI systems that should be considered [19]. Specifically speaking, when dealing with scientific literature, it holds different expectations compared to, for example, English literature. In some of the AI technologies, it was observed that when searching for answers that required a more scientific knowledge base, there were some inconsistencies, and incorrect answers were being generated [19]. There are specific patterns that are relevant to neurology, and these are easily detectable with computer-aided diagnosis (CAD) systems that use AI [20]. It is important to research the different types of applications in which these AI systems can be applied. Most of the benefits listed above are applications in which AI has assisted clinicians and physicians in providing exceptional patient care. One of which is the assistance in clinical decision-making. The CAD systems have a process of specific signaling techniques, and with the use of AI, the signal and image interpretations are much more effectively conducted [20]. 

Once the input is added, it creates a signal transformation, which leads to extraction and dimension reduction [20]. The system has further processes installed that allow it to create the optimal settings, finally classifying it to a certain category and leading to the end diagnosis [20].

The time from onset of symptoms, imaging, diagnosis, and management plays an important role in the patient’s outcome. AI can be used to reduce the time from imaging to diagnosis of various neurological conditions. It can be used for analyzing CT scans, MRI scans, and X-rays. AI-driven algorithms help detect patterns indicative of neurological conditions like Alzheimer’s disease, seizures, Parkinson’s disease, multiple sclerosis, and various brain tumors [21]. Several classes of AI have been studied, including ML and deep learning (DL), both of which utilize artificial neural networks (ANN) inspired by neuronal architecture [22]. ML and DL have been instrumental in the diagnosis of neurological disorders by improving EEG interpretation, pattern recognition, and real-time clinical decision-making. Beyond diagnosis, AI is now being leveraged to develop patient-specific predictive models that estimate prognosis by integrating multimodal data, including neuroimaging, electrophysiology, and clinical variables [22]. These models utilize advanced algorithms such as convolutional neural networks (CNNs) and recurrent neural networks (RNNs) to identify subtle biomarkers that may indicate seizure recurrence, treatment response, and long-term cognitive outcomes. Additionally, AI-driven prognostic models can assess patient trajectories by analyzing large-scale datasets, offering a personalized approach to epilepsy management. Studies have shown that predictive modeling can optimize treatment strategies, reduce misdiagnosis, and enhance individualized patient care, thereby improving overall outcomes in NCS and other neurological conditions [22].

AI in the diagnosis of non-convulsive seizures

AI and ML have advanced epilepsy care by enhancing EEG interpretation and seizure prediction accuracy. Many algorithms have been proposed to analyze EEG [22]. Pattern recognition algorithms, such as wavelet and Fourier transforms, play a key role in identifying seizure-specific waveforms; the wavelet transform provides detailed analysis, while the Fourier transform is efficient for real-time processing [23].

DL algorithms including CNN, RNN, and graph neural networks (GNN) have advanced seizure detection and prediction [24,25]. CNNs excel in detecting abnormal spatial patterns by converting EEG data into spectrograms that capture temporal and spectral dynamics, achieving 88.7% accuracy, 90% specificity, and 95% sensitivity in a study by Acharya et al. [26]. On the other hand, RNN and long short-term memory (LSTM) can identify prolonged or recurring seizures. RNNs are used for cEEG data in seizure prediction and detection, while LSTM is used for time-series data [26]. 

**Figure 1:**
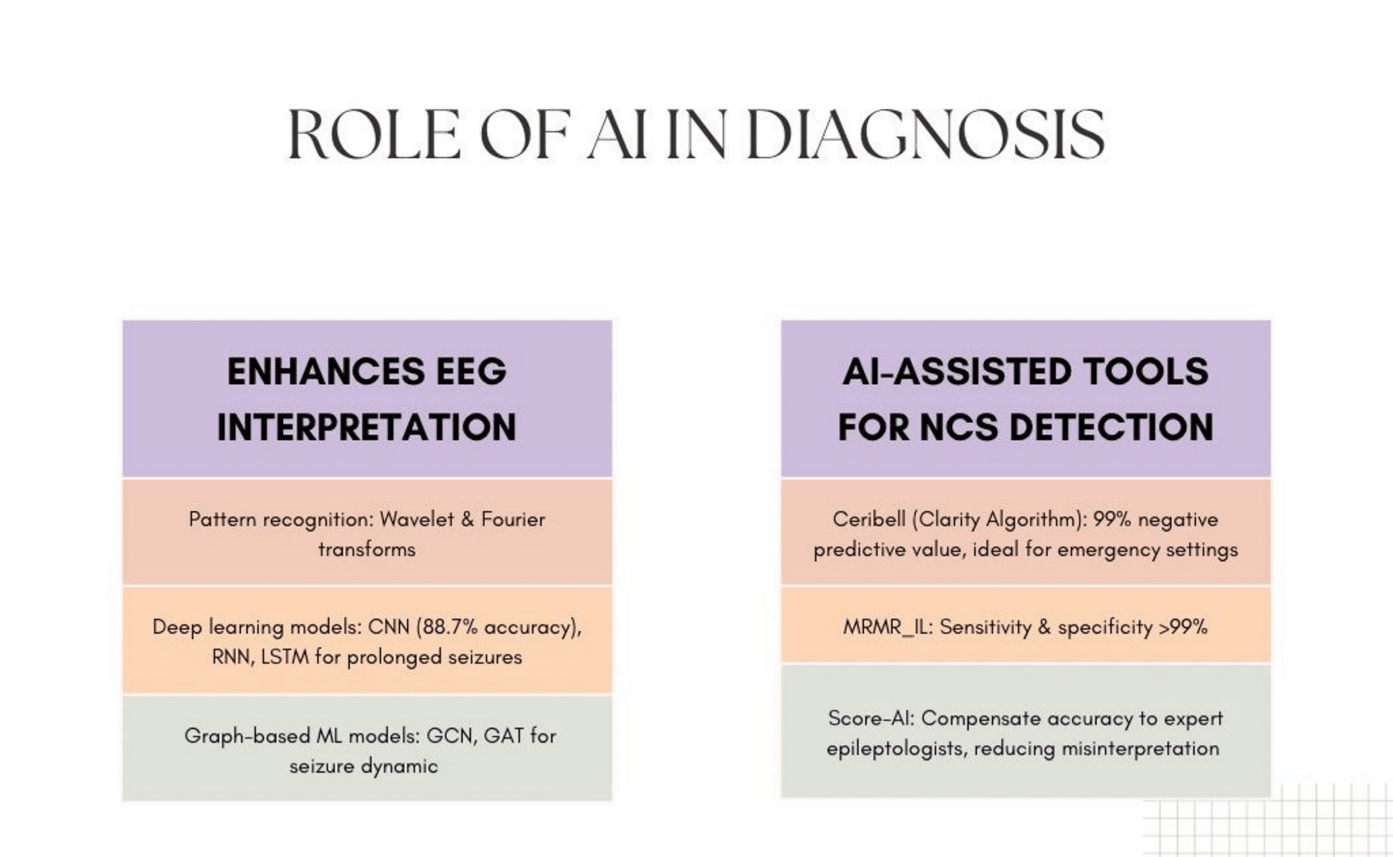
Role of AI in diagnosis

Additionally, graph ML, including graph attention networks (GAT) and graph convolutional networks (GCN), incorporate spatial information from electrode placement to analyze seizure dynamics [25]. A study by Madakadze et al. introduced the Ceribell system (Ceribell, Sunnyvale, CA), featuring an AI algorithm called Clarity that monitors seizure activity within five minutes, making it ideal for emergency settings [6]. Clarity demonstrated a 99% negative predictive value, effectively ruling out and detecting seizures (Figure 1) [6]. 

ML models for seizure detection

Various algorithms are essential to ML, including support vector machines (SVM), k-nearest neighbors (KNN), linear discriminant analysis (LDA), random forests (RF), and gradient boosting machines (GBM) [25,27]. SVM, a popular supervised learning algorithm, identifies an optimal hyperplane to categorize data [25,27]. When paired with wavelet transforms, SVM achieves high temporal and spectral resolution, with studies showing a sensitivity of 99.1% [28]. 

The KNN algorithm classifies EEG segments by comparing them to labeled data, achieving up to 93.64% accuracy, though its computational demands limit real-time use [25]. Furthermore, LDA is a statistical method used for data classification and dimensionality reduction [29]. RF and GBM are ensemble methods that combine multiple weak models to enhance classification. XGBoost, a GBM variant, achieved 94.46% accuracy in seizure detection [25]. RF offers greater stability and has also been effective in classifying conditions such as dementia, mild cognitive impairment, Alzheimer's disease, and psychogenic non-epileptic seizures (ES) [29].

AI algorithms have been used in several clinical trials to detect NCS. Notable among them are Ceribell, MRMR_IL, and SCORE-AI. The Ceribell system, cleared by the Food and Drug Administration, is one of the most studied and extensively implemented algorithms. A study by Kamousi et al. [30] proved Ceribell to have 100% sensitivity for status epilepticus and 88% for highly epileptiform patterns. Furthermore, it has 99% accuracy for ruling out seizures. Studies by Ward et al. [31], Eberhard et al. [32], and Wright et al. [33] determined it to be feasible for implementation in a community hospital setting and/or an academic hospital setting. Few other related studies in various settings such as ICU [34] and emergency settings [35] have all proven its efficacy as well as its role in reducing anti-seizure medications [36].

The MRMR_IL method has been used for the detection of NCS in patients with underlying epilepsy as well as NCS as a consequence of acute brain dysfunction. It has been proven to outperform Hard_IL, Cross_IL, and batch methods with a sensitivity and specificity above 99%, according to Rodríguez Aldana et al. [37].

Another advantage of implementing AI systems has been a decrease in the time to EEG coupled with an increase in the physician’s confidence in the diagnosis as well as the implementation of a timely and improved treatment plan [38]. AI algorithms have also been compared to traditional methods of diagnosis, such as the reading of electroencephalograms by epileptologists, which are time intensive as well as prone to misinterpretation as opposed to AI models like SCORE-AI, which, besides being made available in underserved areas, show diagnostic accuracy comparable to human experts along with excelling in analyzing extensive datasets, identifying subtle patterns and reducing interobserver variability [15].

AI in the management of seizures 

AI-Driven Monitoring Devices in Seizure Management

Epilepsy management is a multi-targeted approach, which includes medication, lifestyle changes, and sometimes surgery [39]. Despite advancements in treatment, about one-third of epilepsy patients continue to experience seizures that are not well-controlled. This significantly impacts the quality of life for both patients and their caregivers [40].

Traditionally, patients relied on self-reporting, which is subjective and inconsistent. Also, EEG monitoring, though useful, often requires hospitalization and continuous observation, making it impractical for long-term use. AI-powered monitoring offers a modern-day equivalent to this, using vast amounts of data and sophisticated algorithms to forecast seizure activity. They use AI algorithms to analyze a variety of patient data, including EEGs, wearable devices like smartwatches or fitness trackers, electronic health records, and patient-reported outcomes based on symptoms or triggers [41,42].

Several AI-powered tools and platforms are revolutionizing epilepsy care. For instance, NeuroPace’s RNS System (NeuroPace, Inc., Mountain View, CA) is an implantable device that monitors brain activity and delivers electrical pulses to prevent seizures [43]. Similarly, Empatica’s Embrace2 (Empatica, Cambridge, MA), a wearable device, uses ML to detect seizure patterns and alert caregivers in real-time [44]. Another innovation, Seer Medical, combines wearable technology with AI to provide continuous monitoring and predictive analytics, enhancing the ability to manage and anticipate seizures effectively [45].

The Internet of Medical Things (IoMT) plays a crucial role in the timely detection of seizures in patients [46]. By utilizing easy-to-wear gadgets and sensory devices, IoMT enables steady monitoring along with real-time data collection. EEG signals and other cardinal information are instantly transmitted to medical professionals, allowing for the rapid recognition of seizure activity. This real-time data transmission supports prompt intervention and improves patient outcomes [46].

IoMT also plays a vital role in facilitating remote patient management, particularly in underserved regions [47]. IoMT-enabled devices allow patients to have monitoring remotely, which minimizes the necessity of regular clinical visits and guarantees prompt medical attention [48]. This study holds significant value within the field of IoMT and epilepsy detection for several reasons. First, it highlights how the technical system of ML can enhance the detection of ES with precision. Second, this study also helps future researchers and developers discover effective ML methods for particular IoMT systems by assessing the execution of various classifiers, which offers crucial knowledge for the selection of algorithms for IoMT applications. Finally, the integration of explainable AI (XAI) methods improves the explicability of model predictions, which is a critical requirement of the medical industry [45-48].

Predictive Analytics in Seizure Management

XAI is an emerging area of research focused on creating algorithms that offer transparent insights into the processes that underlie the decisions and predictions generated by AI. These operations help in providing clarity about how AI arrives at its conclusions, allowing end users to interpret and trust its outputs more effectively [49]. By utilizing extensive patient data, such as medical histories, medical imaging, and laboratory outcomes, XAI can recognize patterns and identify the earliest indicators of illnesses. Additionally, XAI algorithms assist healthcare professionals in pinpointing high-risk patients and devising tailored treatment plans [49-52].

AI’s ability to predict NCS before they occur is a transformative development in epilepsy management. Predictive analytics in this context leverages pre-ictal EEG features to identify subtle changes that precede seizure onset. Several studies have demonstrated that computational models can predict seizures minutes to an hour before they occur, achieving sensitivities of 80-90% [51]. These predictions are critical for timely interventions, potentially preventing adverse outcomes such as neuronal injury or prolonged hospital stays.

Modern approaches emphasize the use of patient-specific models to refine prediction accuracy. DL algorithms like CNNs and LSTMs are particularly promising in this regard. CNNs are adept at analyzing spatial patterns in EEG data, while LSTMs excel at handling temporal sequences, making them highly suitable for seizure prediction tasks [52]. Furthermore, research is increasingly focusing on integrating multimodal data, such as combining EEG features with other clinical parameters, to enhance model robustness and reliability.

Ongoing studies are also exploring ways to make AI-driven predictions more actionable for clinicians. For instance, predictive models coupled with wearable devices can provide real-time alerts, allowing patients and caregivers to prepare for potential seizures. These advancements highlight the potential for AI to revolutionize seizure management by not only predicting seizures with greater accuracy but also enabling proactive care strategies to improve patient outcomes. Future research should continue to refine these models, addressing challenges such as generalizability across diverse patient populations and minimizing false positives to build trust in clinical applications.

AI Prediction in Surgical Management 

ML algorithms have demonstrated potential in identifying suitable candidates for surgery and predicting surgical outcomes, especially following temporal lobectomy. Grigsby et al. utilized an ANN algorithm to predict the outcomes of anterior temporal lobectomy by analyzing clinical, electrographic, neuropsychological, imaging, and surgical data from 65 patients. Their approach achieved a sensitivity of 80.0% and specificity of 83.3% for predicting Engel I outcomes and 100% sensitivity and 85.7% specificity for combined Engel I or II outcomes [50].

Personalized Treatment Plans Using AI

Epilepsy treatment outcomes vary based on intrinsic characteristics, brain lesions, and extrinsic factors. Personalized approaches and optimal treatment methods are crucial for accurate diagnosis and management [53]. Computational studies on epilepsy utilize high-performance computing technologies and mathematical algorithms to analyze large datasets, providing automated protocols for clinical decision-making and guiding future research in personalized medicine [54].

**Figure 2:**
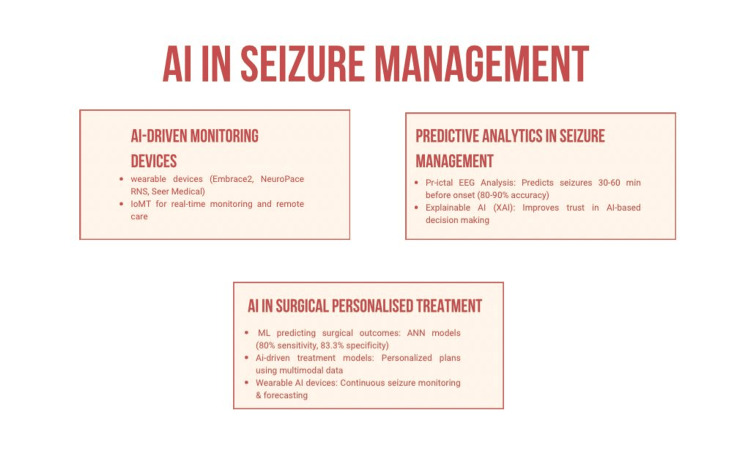
AI in seizure management

AI-driven tools are increasingly being integrated into patients' daily lives to enable continuous monitoring and personalized care. Wearable AI devices, such as multimodal wristbands and subcutaneous EEG systems, provide real-time seizure detection and forecasting, allowing for timely interventions and enhancing patient safety [44,54]. For instance, the Empatica Embrace wristband, equipped with ML-based algorithms, uses accelerometry and electrodermal activity sensors to detect seizure-like events and alert caregivers in real-time, reducing response delays and improving outcomes for high-risk patients [44]. Similarly, ultra-long-term wearable EEG devices offer a promising solution for continuous seizure monitoring, enabling objective seizure burden assessments and facilitating treatment adjustments based on real-world data [54]. These advancements not only improve seizure detection accuracy but also empower patients to manage their condition more effectively by integrating AI into their daily routines. Future research should focus on refining these technologies to minimize false positives, enhance patient comfort, and expand access to AI-driven epilepsy management in underserved regions (Figure 2).

Advancements in AI and ML for Personalized Healthcare

ML is a key AI technique that combines statistics and computer science to improve performance through data analysis. It is categorized into supervised learning, which uses labeled data for classification or regression, and unsupervised learning, which uses unlabeled data for clustering or association. DL, a subset of ML using ANN, is particularly advantageous for discovering features in data. Variants like convolutional CNN for image data and RNN for time-series data have been widely employed [55,56].

Biophysical modeling approaches, such as neural network modeling, replicate neural dynamics to investigate brain functions and dysfunctions. These include bottom-up models (microscale, focusing on neurons and synapses) and top-down models (macroscale, using brain connectomes). Personalized brain network modeling employs neural mass models derived from structural imaging data, optimizing surgical strategies and predicting therapeutic outcomes for specific diseases [57-60]. Additionally, web-based decision support systems like EpiPick guide antiepileptic drug selection, enhancing treatment outcomes by reducing side effects and improving seizure control [60].

AI integration in healthcare enables personalized treatment through big data analysis and predictive modeling, improving diagnostic accuracy, treatment planning, and cost efficiency. This approach enhances patient outcomes, optimizes resource use, and facilitates precision medicine while addressing ethical and privacy concerns. Continuous innovation and collaboration are essential for AI’s effective integration into healthcare, ensuring improved patient care and a global transformation in healthcare delivery [61-64].

Challenges and ethical considerations 

Patient confidentiality is a major concern in AI healthcare applications, as de-identified datasets can potentially be re-identified through data triangulation, risking privacy breaches [65]. Advancements in technology and cybersecurity attacks on electronic records further amplify these risks. Additionally, bias in AI systems can disproportionately affect underrepresented populations due to systemic tendencies in algorithm training [66]. Addressing these biases is essential to uphold ethical principles like justice and fairness, with frameworks such as FAIR offering guidance [67].

Effective implementation of AI in healthcare requires a multidisciplinary approach. Collaboration between neurologists, AI specialists, data scientists, and healthcare administrators is crucial to ensure smooth integration and adoption in clinical practice. Neurologists bring clinical expertise to define practical applications, while AI specialists and data scientists develop and refine algorithms to address specific challenges. Healthcare administrators play a vital role in managing resources, setting policies, and ensuring compliance with ethical standards. This collaborative effort can enhance the reliability, usability, and acceptance of AI technologies in healthcare settings.

Legal accountability is another challenge, as AI lacks legal status, leaving humans responsible for any harm caused by inaccurate outputs. Clear regulations are needed to define liability and safeguard users [68]. Furthermore, AI's role in healthcare could disrupt doctor-patient relationships if outcomes are misunderstood. Building trust-encompassing self-trust, interpersonal trust, and system trust requires healthcare professionals to enhance their expertise in digital technologies and demonstrate their benefits in patient care (Table 1) [69,70].

**Table 1:** Benefits and limitations of use of AI-driven technology

Benefits	Limitations
Use of AI results in improved EEG interpretation, reducing human errors and interobserver variability.	AI raises concerns about patient data security, informed consent, and misuse of personal health information.
Using machine learning enables the analysis of patient data for early seizure detection.	Potential biases in training datasets may lead to disparities in healthcare outcomes.
Wearable AI devices enable continuous monitoring and timely alerts to caregivers.	Lack of clear legal status for AI decision-making raises liability and accountability issues.
AI tailors treatment based on patient-specific factors, optimizing seizure management.	Requires collaboration between neurologists, AI specialists, and healthcare policymakers for successful implementation.
AI reduces clinician workload through AI-driven automation in data interpretation and imaging.	Patients may be hesitant to trust AI-driven systems, requiring increased transparency and education.

Future directions 

The future of AI in NCS detection involves rapid advancements in technology, early detection potential, collaborative research efforts, and essential clinician education. ML and DL are transforming digital healthcare, with federated learning (FL) standing out as a newer approach. FL offers ML model training without sharing sensitive data, guaranteeing privacy and unbiased models [71]. Additionally, brain-machine interfaces (BMIs) are becoming important tools for diagnosing neurological conditions such as Parkinson’s disease and stroke, contributing to the progress of brain diagnostics and neuroimaging techniques [72]. The successful implementation of AI in healthcare depends on the collaboration between healthcare providers, researchers, and AI professionals. Healthcare providers contribute clinical knowledge and disease expertise, enhancing the precision of AI models.

Meanwhile, researchers and AI professionals provide technical skills and AI tool recommendations that aid with diagnosis, early disease detection, and workload management [72]. Educational programs are crucial for providing clinicians with the necessary skills to understand and apply AI tools effectively. Targeted training helps healthcare providers integrate AI into their practice, improving diagnostic accuracy and patient care. Incorporating AI models into routine EEG monitoring could facilitate the early identification of NCS, allowing for preventive measures to be taken before serious complications arise. Further research should investigate how personalized analyses and enhanced network metrics could optimize epilepsy detection and treatment approaches [73].

## Conclusions

AI has revolutionized the diagnosis and management of NCS through advancements in real-time monitoring, predictive analytics, and personalized treatment strategies. Wearable and subcutaneous EEG devices, along with ML models, have improved seizure detection and prognosis, while AI-driven decision support systems have optimized clinical workflows. Despite these promising applications, challenges such as patient data privacy, algorithmic bias, and regulatory oversight must be addressed to ensure ethical and equitable AI integration. Emerging technologies like FL and BMIs offer new opportunities to enhance AI’s role in epilepsy care. Moving forward, interdisciplinary collaboration and robust regulatory frameworks will be critical in translating AI innovations into widespread, patient-centered clinical practice.
